# Infection Rate of SARS-CoV-2 in Asymptomatic Healthcare Workers, Sweden, June 2022

**DOI:** 10.3201/eid2810.221093

**Published:** 2022-10

**Authors:** Kim Blom, Sebastian Havervall, Ulrika Marking, Nina Greilert Norin, Philip Bacchus, Ramona Groenheit, Andreas Bråve, Charlotte Thålin, Jonas Klingström

**Affiliations:** Karolinska Institutet, Stockholm, Sweden (K. Blom, S. Havervall, U. Marking, N. Greilert Norin, C. Thålin, J. Klingström);; Public Health Agency of Sweden, Solna, Sweden (K. Blom, R. Groenheit, A. Bråve, J. Klingström);; Karolinska Institutet Danderyd Hospital, Stockholm (S. Havervall, U. Marking, N. Greilert Norin, C. Thålin);; Swedish Armed Forces, Umeå, Sweden (P. Bacchus)

**Keywords:** COVID-19, SARS-CoV-2, healthcare workers, Sweden, coronavirus disease, severe acute respiratory syndrome coronavirus 2, viruses, respiratory infections, zoonoses, vaccine-preventable diseases

## Abstract

Given the recent surge in SARS-CoV-2 Omicron infections, we performed a quantitative PCR screening survey during June 28–29, 2022, in Stockholm, Sweden, to investigate SARS-CoV-2 point prevalence in a group with high exposure risk. Results showed SARS-CoV-2 infection in 2.3% of healthcare workers who were asymptomatic at time of sampling.

Emerging data show a rapid increase in the prevalence of SARS-CoV-2 infection linked to an increase in COVID-19 cases, which is being driven by the SARS-CoV-2 Omicron variant. Compared with previous variants, Omicron has shown superior capacity for transmission and less sensitivity to neutralizing antibodies induced by vaccination or prior infection with other variants of the virus (*1*). Initially, the Omicron sublineages BA.1 (including BA.1.1) and BA.2 spread globally at a rapid pace, infecting a large proportion of the population, including vaccinated persons. Nonetheless, vaccines have been shown to provide good protection against severe disease (*2*). Recently, 2 new sublineages of Omicron, BA.4 and BA.5, have emerged (*3*). These variants show an even stronger capacity to elude infection- and vaccine-induced immune responses, even evading antibodies in serum from BA.1-infected persons (*4*,*5*). Such findings raise concerns that a high community spread might lead to an increasing number of severe cases and a subsequent surge in global hospitalization rates. We performed a quantitative real-time PCR (qPCR) screening survey to estimate the point prevalence of SARS-CoV-2 infection among asymptomatic (defined as having no symptoms at time of sampling) healthcare workers at Danderyd Hospital, Stockholm, Sweden, during June 28–June 29, 2022.

In April and May of 2020, the COMMUNITY study enrolled 2,149 healthcare workers employed at Danderyd Hospital (*6*). Once enrolled, study participants provided blood samples every 4 months for SARS-CoV-2 serologic assessment (*7*). Information regarding vaccination status was obtained through the Swedish vaccination register (VAL Vaccinera), and SARS-CoV-2 infection was determined by either seroconversion before vaccination or positive PCR test results obtained from the national communicable diseases register, SmiNet (Public Health Agency of Sweden).

We conducted a qPCR screening survey during June 28–June 29, 2022. We invited all COMMUNITY-study participants who had provided a blood sample in January 2022 (n = 1,412) to participate in the screening survey via a mobile application program. We restricted participation in the survey to healthcare workers who were actively working and who had been asymptomatic for >5 days before screening. We gathered self-administered naso-oropharyngeal/saliva swab specimens (*8*), which were collected at Danderyd Hospital during work hours, and transported those samples to the National Pandemic Center in Stockholm for assessment by qPCR. The screening survey was approved by the Swedish Ethical Review Authority (dnr 2020–01653) and conducted in accordance with the declaration of Helsinki. We obtained written informed consent from all survey participants.

A total of 259 healthcare workers (18.3% of all invited participants) with no symptoms at the time of inclusion underwent qPCR screening. A large proportion (88%) of participants had received 3 vaccine doses, and 50% had been confirmed as having 1 (46%) or 2 (4%) prior SARS-CoV-2 infection(s) (Table). In total, 6 participants (2.3% [95% CI 1.1%–5.0%]) tested positive by qPCR screening; 5 had received 3 vaccine doses, and 2 had a confirmed previous SARS-CoV-2 infection (Table). Just 1 of the 6 participants who tested positive was unvaccinated and previously uninfected. Five samples could be successfully sequenced, revealing 1 infection traced to the BA.2.9.2 sublineage and 4 infections traced to BA.5 (BA.5.1 [2 cases], BA.5.2, and BA.5.3), suggesting community spread of several variants of Omicron. Isolation on A549-ACE2 cells was successfully accomplished for 2 samples.

**Table:** Characteristics of 259 asymptomatic HCWs who participated in a quantitative real-time PCR screening survey, Stockholm, Sweden, June 28–29, 2022*

Characteristic	All HCWs		Infected HCWs
Total	259 (100)		6 (2.3)
Sex				
M		26 (60)	1 (17)	
F		233 (40)	5 (83)	
Median age, y		51	48	
Vaccination status				
No vaccination		5 (2)	1 (17)	
1 vaccine dose		2 (1)	0	
2 vaccine doses		24 (9)	0	
3 vaccine doses		228 (88)	5 (83)	
Previous Infections				
1 infection		119 (46)	2 (33)	
2 infections		11 (4)	0	

*Values are no. (%) except as indicated. HCW, healthcare worker.

A 2.3% point prevalence of SARS-CoV-2 infection among asymptomatic healthcare workers indicates widespread transmission of SARS-CoV-2. This prevalence aligns with estimates from the United Kingdom (*9*), where ≈1 in 30 persons was estimated to be infected by SARS-CoV-2 on July 1, 2022. A recent survey conducted in March 2022 during the BA.1/BA.2 wave estimated an overall prevalence of SARS-CoV-2 infection in Sweden of 1.4% (*10*). Although our survey differs in design from that earlier survey, results of both indicate a trend of increased circulation of variants in the population of Sweden, despite the summer season, high vaccine coverage, and a high rate of prior infection. 

Additional PCR screenings of our cohort, conducted before the survey we report, revealed that ≈10% of SARS-CoV-2-infected participants remained asymptomatic over the course of the infection (*8*). In parallel with the testing on June 28–29, we performed a substudy using the same cohort during the same days to attempt to isolate the BA.5 sublineage from participants diagnosed with COVID-19 within the previous 5 days. Ten participants were included, and the BA.5 variant of the virus could be isolated on A549-ACE2 cells in 5 samples. Ten people is likely an underrepresentation of true cases in this cohort, but these findings show nonetheless that at least 0.7% of the healthcare workers were diagnosed with COVID-19 at the same time as an additional 2.3% of the healthcare workers had an asymptomatic infection. 

We theorize that the latest surge in SARS-CoV-2 infection, in Sweden and elsewhere, can be likely explained by the emergence of the BA.5 variant. The observed prevalence of 2.3% in asymptomatic healthcare workers in Sweden implies a need to take precautions to protect this high-risk population, in hospitals and all other vulnerable settings.
